# Influence factor research on deacidification process for high carbon content gas field by numerical simulation—a case study of the Oudeh gas field

**DOI:** 10.1186/s40064-015-1461-1

**Published:** 2015-11-06

**Authors:** Yitang Lv, Kun Huang, Cheng Huang, Xiaohui Lu

**Affiliations:** School of Petroleum Engineering, Southwest Petroleum University, Chengdu, Sichuan China; Key Laboratory of Integrated Regulation and Resources Development of Shallow Lakes of Ministry of Education, School of Earth Science and Engineering, Hohai University, Nanjing, Jiangsu China

**Keywords:** High carbon contents, Deacidication process, Optimization of parameters, Input temperature of lean solution, Reflux ratio, Number of trays, Oudel gas field

## Abstract

**Introduction:**

High concentrations of CO_2_ in natural gas affect its calorific value and corrode the equipment and pipelines related to its transportation and usage. Therefore, strict control over the H_2_S and CO_2_ contents in natural gas is essential. CO_2_ is an important industrial gas that can bring a great deal of economic profit when it is fully utilized.

**Case description:**

The natural gas produced at the Oudeh gas field in Syria contains high carbon content natural gas, in which the CO_2_ content is in the range of 17.5–18.8 %, while the H_2_S content is in the range of 2.8–3.2 %. However, there have been few studies conducted on treatment solutions for natural gas with high carbon contents. In this paper, several commonly used methods for deacidification of natural gas were introduced. Among these methods, the most suitable one was chosen for desulfurization and decarbonization of the natural gas produced at the Oudeh gas field based on its gas quality.

**Conclusions:**

Optimization and analysis of the primary operating parameters for the desulfurization and decarbonization processes were conducted to obtain the optimized values for the input temperature of the lean solution (42 °C), reflux ratio (0.8), number of trays in the absorber unit (17) , and circulation rate of the lean solution (330 m^3^/h), etc. Additionally, the influence of the operating pressure of the regenerator unit on the regeneration system was also investigated. The energy consumption of the apparatus and the corrosion level of sour gas to the apparatus were reduced after optimization. Based on the investigation of the natural gas treatment for this gas field, it can serve as a reference for the purification of high carbon contents natural gas.

## Background

There is a burgeoning demand for natural gas in industry because it is a clean fuel. It has been predicted that the world will enter a “natural gas era” in the middle of the 21st century (Zhang 1997). A series of treatments is required for natural gas to be extracted from underground before it can be commercialized. Different treatments are needed for natural gases with different natures. “Acid gas” refers to natural gas containing H_2_S, CO_2_, and RSH (organic sulfur). H_2_S is highly toxic and burns into SO_2_, causing air pollution and cracking of pipes after severe corrosion. High levels of CO_2_ directly affect the calorific value of natural gas and corrode the system and pipelines installed for its transportation and usage. Therefore, the levels of H_2_S and CO_2_ in natural gas have to be strictly controlled. The quality of purified natural gas must meet the second standard of China’s quality specification for natural gas, in which the content of H_2_S must be less than 7 mg/m^3^ and the content of CO_2_ must be less than 3 %.

In 1880s, the American company Union Carbide is the pioneer company formulated the MDEA solution, Ucarsol HS, and further developed it into a series of products. Thereafter, formulated CR solutions, primarily used for CO_2_ removal, were also developed (Yu 1985). The Dow chemical company based in the United States also developed similar formulated solutions such as the Gas/Spec SS and CS series solutions. In the 1950s, German companies Lurgi GmbH and Linde AG jointly developed low-temperature methanol absorption technology. Owing to its excellent performance, it was widely used in urban gas as well as the oil and gas industries. Currently there are over 80 installations operating low-temperature methanol absorption technologies worldwide (Niu et al. 2003; Hoochgesand 1970). In 1965, American company Allied developed the Selexol solution (polyethylene glycol dimethyl ether) as a physical absorption method that was used for the removal of CO_2_ from natural gas starting in the early 1980s. Recently, the Selexol solution has been widely employed for the removal of H_2_S, CO_2_, COS, sulfides, mercaptans, and other hazardous components from natural gas, fuel gas, and syngas (Qin et al. 2008; Cheng et al. 2002). The ARI Company successfully developed the LO-CAT process used for catalytic liquid-phase oxidation–reduction desulfurization using iron-based catalysts. It is suitable for the purification of natural gas with 0.2 ~ 10 T/d sulfur content. The difference between the LO-CAT and oxyamine desulfurization processes is that the former is an oxidation–reduction process for the removal of H_2_S but does not remove CO_2_. Since the successful development of the LO-CAT desulfurization process in the 1980s, as many as 232 installations have been set up worldwide including those that have been completed and those under construction as of 1998 (Xu et al. 2004). In the early 1980s, the Monsanto Company developed membrane separation technology for the removal of CO_2_ from natural gas. However, the application of this technology was hindered by the high production cost of the membrane (Wang et al. 1999; Long and Long 1993).

In China, the oxyamine process is the most commonly used method for the desulfurization and decarbonization of natural gas. In the 1980s, Nanjing Chemical Industry Company successfully developed an absorption solution called NHD that resembled the physical and chemical properties of Selexol, which was later developed into a NHD purification process. The NHD process is currently widely used for desulfurization and decarbonization purposes in small fertilizer plants, ammonia plants, and methanol plants (Hoochgesand 1970). Since 1993, the research institute of PetroChina Southwest Oil and Gasfield has been cooperating with Zhejiang Zhenhai Petrochemical Engineering General Plant in an effort to substitute MEA for a MDEA method in order to reduce the energy consumption of vapor by 40 %. Until now, pure MDEA or formulated solvents with a mixture of MDEA and other physical solvents and chemical additives have been used in most purification installations for natural gas and gas refineries in China (Chen 2003; He et al. 2009).

## Mathematical model

HYSYS is a simulation software developed by Hyprotech Company based in Canada that plays a dominant role in simulation techniques in the petrochemical industry around the world. It is also used in wide range of fields, including petroleum extraction, storage and transportation, petrochemical industries, natural gas processing, fine chemical industries, refining industries and pharmaceutical industries.

### Vapor–liquid equilibrium

The calculation of liquid phase equilibrium is essential to every operating unit. Therefore, any variation in temperature, pressure, or composition may cause changes in the initial equilibrium system until it reaches a new equilibrium state. The (ϕ–γ) method is used for the calculation of vapor–liquid equilibrium.

$$K = \frac{{y_{i} }}{{x_{i} }} = \frac{{\gamma_{i} f_{i}^{o} }}{{\phi_{i}^{V} P}}$$

where *K* is the phase equilibrium constant; $\gamma_{i}$ is the fugacity coefficients of *i* component in liquid phase; $f_{i}^{o}$ is the fugacity of *i* component in liquid phase under standard conditions; $\phi_{i}^{V}$ is the fugacity coefficients of i component in vapor phase.

### Balance equation of acid gas in the amine solution

The balance equation in a H_2_S-CO_2_-Am-aqueous system involves seven chemical equations, two Henry’s Law, three mass-balance equations, and one electroneutrality equation.

#### Reaction equations

$$\begin{aligned} AmH^{ + } \Leftrightarrow Am + H^{ + } \hfill \\ \hfill \\ \end{aligned}$$

$$K_{1} = \left[ {Am} \right]\left[ {H^{ + } } \right]/\left[ {AmH^{ + } } \right]$$

$$AmCOO^{ - } + H_{2} O \Leftrightarrow Am + HCO_{3}^{ - }$$

$$\begin{aligned} \hfill \\ K_{2} = \left[ {Am} \right]\left[ {HCO_{3}^{ - } } \right]/\left[ {AmCOO^{ - } } \right] \hfill \\ \end{aligned}$$

$$\begin{aligned} CO_{2} + H_{2} O \Leftrightarrow H^{ + } + CO_{3}^{ - } \hfill \\ \hfill \\ \end{aligned}$$

$$K_{3} = \left[ {H^{ + } } \right]\left[ {HCO_{3}^{ - } } \right]/\left[ {CO_{2} } \right]$$

$$\begin{aligned} H_{2} O \Leftrightarrow H^{ + } + OH^{ - } \hfill \\ \hfill \\ \end{aligned}$$

$$K_{4} = \left[ {H^{ + } } \right]\left[ {OH^{ - } } \right]$$

$$HCO_{3}^{ - } \Leftrightarrow H^{ + } CO_{3}^{2 - }$$

$$\begin{aligned} \hfill \\ K_{5} = \left[ {H^{ + } } \right]\left[ {CO_{3}^{2 - } } \right]/\left[ {HCO_{3}^{ - } } \right] \hfill \\ \end{aligned}$$

$$H_{2} S \Leftrightarrow H^{ + } + HS^{ - }$$

$$\begin{aligned} \hfill \\ K_{6} = \left[ {H^{ + } } \right]\left[ {HS^{ - } } \right]/\left[ {H_{2} S} \right] \hfill \\ \end{aligned}$$

$$HS^{ - } \Leftrightarrow H^{ + } + S^{2 - }$$

$$\begin{aligned} \hfill \\ K_{7} = \left[ {H^{ + } } \right]\left[ {S^{2 - } } \right]/\left[ {HS^{ - } } \right] \hfill \\ \end{aligned}$$

where $K_{1}$ is the equilibrium constant, *i* is the reaction number, [CO_3_^2−^] and etc. are the concentrations of ions or compounds in the solution.

#### Henry’s law

$$P_{c} = H_{c} \left[ {CO_{2} } \right]$$

$$P_{s} = H_{s} \left[ {H_{2} S} \right]$$

where P_s_, P_c_ is the partial pressure of H_2_S and CO_2_, respectively, kPa; H_s_, H_s_ are the Henry’s constant of H_2_S and CO_2_, respectively, kPa L/mol.

#### Electroneutrality

$$\left[ {HCO_{3}^{ - } } \right] + \left[ {OH^{ - } } \right] + 2\left[ {CO_{3}^{2 - } } \right] + \left[ {HS^{ - } } \right] + 2\left[ {S^{2 - } } \right] + \left[ {AmCOO^{ - } } \right] = \left[ {AmH^{ + } } \right] + \left[ {H^{ + } } \right]$$

#### Mass-balance equation

$$m = \left[ {AmCOO^{ - } } \right] + \left[ {AmH^{ + } } \right] + \left[ {Am} \right]$$

$$m\alpha_{c} = \left[ {HCO_{3}^{ - } } \right] + \left[ {CO_{3}^{2 - } } \right] + \left[ {CO_{2} } \right] + \left[ {AmCOO^{ - } } \right]$$

$$m\alpha_{s} = \left[ {HS^{ - } } \right] + \left[ {S^{2 - } } \right] + \left[ {H_{2} S} \right]$$

where m is the concentration of amine, mol/L; α_c_ and α_s_ are the equilibrium solubility of CO_2_ and H_2_S in amine, respectively, mol/mol.

## Case study

### Project profile

The natural gas investigated in this paper was high carbon content natural gas extracted from the Oudeh gas field in Syria (Belongs to Oudeh oilfield, the owner of the land gave permission to conduct the study on this site. (1) I state that no specific permissions were required for these locations/activities. (2) I confirm that the field studies did not involve endangered or protected species). The composition of its feed gas was illustrated in Table 1.

**Table 1:** The composition of feed gas

Composition	Content (%)
CH_4_	78.0
C_2_H_6_	0.8
C_3_H_8_	0.2
CO_2_	18.0
H_2_S	3.0

The processing capacity of the installment, flow rate, pressure (absolute pressure), and temperature of the feed gas were set as 250 × 10^4^ Nm^3^/d, 230 × 104, 7.5 MPa, and 25 °C, respectively.

The quality of the natural gas has to be taken into consideration when choosing the most suitable method for desulfurization and decarbonization processes. For example, the oxidation–reduction method is mainly used for the removal of H_2_S and is unsuitable for systems with CO_2_ content. The CO_2_ content of the purified gas does not reach the specification of being less than 3 % after undergoing the membrane separation process. In addition, it is highly unstable, and generates high production costs (Ren and An 2005). The physiochemical absorption method has only found success in small installations but it is still not stable enough to be widely used (Gupta 1986). The simulation result shows that 39.1 % of methane will be lost using the Selexol method, showing a lack of economic benefit. Compared to those methods, the chemical absorption method seems to be the most suitable desulfurization and decarbonization process to purify natural gas with high CO_2_ content.

During the deacidification process, monoethanolamine (MEA) in the solution degrades CO_2_ to produce some non-renewable substances, which results in partial loss of its desulfurization capability. Hence, this method is not being selected. Methyldiethanolamine (MDEA) is highly selective towards H_2_S, is less corrosive, requires low energy consumption for regeneration, and has wide applications. Although this method is applicable for H_2_S removal, this study primarily dealt with the removal of CO_2_, and hence, this method was also unsuitable (Xue 1995). In the F1exsorb PS process, the activated MDEA and sterically-hindered amines are suitable for use with natural gas with high carbon content. However, its high cost has hindered its wide applications. Compared to MEA, diethanolamine (DEA) has relatively high solubility, lower degradability, is less corrosive, and is not selective towards either H_2_S or CO_2_. It is suitable to be used in natural gas, manufactured gas, and feed gas with high contents of organic sulfides. Thus, it was selected for this system.

It is known that feed gas contains high CO_2_ content. Although the MDEA solution possesses a number of advantages, it has a slower reaction rate in the removal of CO_2_. To overcome this problem, a certain amount of DEA (MEA) was mixed with the MDEA solution to improve the absorption rate of CO_2_. As a result, the MDEA-DEA mixed-amines solution exhibited a faster absorption rate, higher CO_2_ removal efficiency, and lower circulation rate of lean solution (Li and Zhao 2009; Wang and Wang 2003). After comparing the simulations of solutions with different ratios and the DEA method, a mixed-amines solution with a ratio of 43 %(w) MDEA + 12 %(w) DEA was chosen as the absorber for the desulfurization and decarbonization processes after purification efficiency, economic benefits, and recycling issues were taken into account.

### Numerical simulation method validation

Based on the field test data provided by the literature (Ma et al. 2012), a set of decarbonization device were put into production on 2009.12 at Changling gas field, with a operation capacity of 120 × 10^4^m^3^/d. The mixed-amines solution is 45 %(w) MDEA + 5 %(w) Activator + 50 %(w) Water, the CO2 content in processed natural gas is 24 % on average in the test.

A numerical simulation is conducted on the base of this test, as presented in Table 2.

**Table 2:** Data comparison between the result of test and numerical simulation

Items	CO_2_ content in purified gas (%)	Lean solution circulation rate (m^3^/h)
Test	2.42	392
Numerical simulation	2.33	384
Relative error	−3.72 %	−2.04 %

A relative error of −3.72 % and −2.04 %, shows fairly good agreement with the test results. It also indicates that the simulation model built by HYSYS yields highly reliable results

### Numerical simulation

In numerical simulation, the process was designed as follows.

The feed gas (F1) was firstly introduced into the horizontal separation to remove impurities and water droplets (F2) that had been carried along with the feed gas (F1). The feed gas (F1) entered the bottom of the absorber unit after the filtration and separation processes. The up-flowing feed gas eventually had countercurrent contact with the down-flowing 55 % (w) mixed-amines lean solution that entered from the top of the tower. Most of the H_2_S and CO_2_ were absorbed by the lean solution here. The wet purified gas (F2) left the tower and subsequently entered the vertical separator for removal of the remaining alcohol amine droplets (F4) before leaving the installation. The wet purified gas (F5) was then transferred into the molecular sieve and dehydration unit. The H_2_S and CO_2_ contents of the wet purified gas (F5) were the contents after being purified.

The DEA-rich solution (F6) that exited from the bottom of the absorber unit was introduced into the flash drum. Hydrocarbon gasses that were dissolved in the solution were released after undergoing the flash evaporation. In the scrubbing section, the up-flowing flash gas (F7) had countercurrent contact with the down-flowing mixed-amines lean solution to remove a portion of the CO_2_ and H_2_S content from the flash gas. After its pressure was regulated, the flash gas (F7) eventually entered the fuel gas system of the purification plant.

The mixed-amines-rich solution (F8) coming from the bottom of the flash drum passed through the filtration system to purge the degradation products and mechanical impurities that had been dissolved in the rich solution. After that, the mixed-amines-rich solution (F9) entered the heat exchanger and was warmed by the lean solution (F10) coming out from the bottom of the regenerator unit. Then, the rich solution (F11) re-entered the regenerator unit from the top and had countercurrent contact with the up-flowing vapor, leading to further desorption of the H_2_S and CO_2_ gases to achieve regeneration of the solution. A reboiler at the bottom of the tower provided the heat for regeneration. After passing through the heat exchanger, the warm mixed-amines lean solution (F12) was removed from the bottom of the regenerator unit and cooled by the rich solution. It (F12) then entered the air cooler and cooler to be further chilled. Subsequently, the lean solution (F13) was transferred back to the MDEA-DEA absorber unit by means of a circulating pump to complete the cycle.

The acidic components and water vapor (F14) leaving from the top of the regenerator unit entered the condenser, and a substantial amount of water vapor was condensed. The acidic condensate (F15) being separated out was directed back to the top of the regenerator unit to recycle the alcohol amines vapor that was carried along by the acidic airflow. The acid gas (F16) was transferred to the sulfur recovery unit or incinerator.

Parameters of the simulated process are shown in Tables 3, 4.

**Table 3:** Parameter for the absorber and regenerator units

Apparatus	Pressure (top) (kPa)	Pressure (bottom) (kPa)	Temperature (top) (°C)	Temperature (bottom) (°C)	Number of trays	Reflux ratio
Absorber unit	7150	7500	40	60	16	–
Regenerator unit	180	200	120	200	25	0.9

**Table 4:** Parameter for the apparatus used in the process

Apparatus	Flash drum	Mixer	Heat exchanger	Air cooler	Cooler	Temperature of outlet pump
Internal pressure of the apparatus (kPa)	0	Balance calculation	50	50	30	–
Other setting	–	–	–	–	–	41 °C

**Fig. 1:**
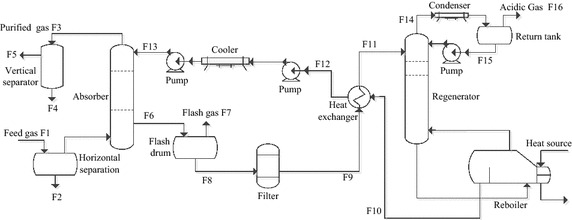
Schematic flow diagram of the deacidification process using mixed-amines solution

The flow diagram of the process is shown in Fig. 1.

## Results

The mass flow parameters of the process using 43 %(w) MDEA + 12 %(w) DEA calculated by the simulation software are shown in Table 5.

**Table 5:** Simulated results of the mass flow parameters of the deacidification process using the mixed-amines solution

Mass flow parameters		Feed gas	Purified gas	Acid gas	Lean solution entering the absorber unit	Rich solution existing the absorber unit	Lean solution existing the regenerator unit	Rich solution entering the regenerator unit	Mixer
Temperature, °C		25	45.7	53.9	41.9	76.7	125.2	81.1	70
Pressure, kPa		75,000	7150	130	7150	7500	200	190	150
Flow rate, kmol/h		4250	3440	799.8	10,080	10,890	9974	10,770	110
Composition mol %									
CO_2_		18	2.4359	73.9106	0.0393	6.2923	0.0406	5.5244	–
H_2_S		3	0.0004	14.4838	0.0060	1.1946	0.0063	1.0811	–
H_2_O		–	0.1791	11.5959	84.0123	77.6971	83.8442	78.4808	–
DEA		–	–	–	3.8303	3.5454	3.8708	3.5835	–
MDEA		–	0.0001	–	12.1116	11.2094	12.2381	11.3296	–
CH_4_		78	96.1459	0.0096	–	0.0787	–	0.0007	–
C_2_H6		0.0800	0.9867	0.0001	–	0.0006	–	–	–
C_3_H_6_		0.0200	0.2468	–	–	0.0001	–	–	–

As can be seen in Table 5, the CO_2_ and H_2_S contents of the purified gas after undergoing the deacidification process using mixed-amines solution were 2.44 and 0.0004 %, respectively, meeting the quality specifications for purified gas. The heat load of the apparatus used during the process is illustrated in Table 6.

**Table 6:** Heat load of the apparatus

Apparatus	Inlet temperature, °C	Outlet temperature, °C	Flow rate	Heat load, kw
Reboiler	129.8	Balance calculation	50	50
Heat exchanger	(Rich solution) 72.3 (Lean solution) 125.2	(Rich solution) 90.0 (Lean solution) 98.6	(Rich solution) 10,770 kmol/h (Lean solution) 9974 kmol/h	9239
Cooler	80.0	40.2	10,080 kmol/h	12,475

## Discussions

During the simulated calculations for the desulfurization and decarbonisation process of natural gas, most of the parameters were derived from empirical or assumed values. Therefore, this paper investigated the input temperature of the lean solution, the reflux ratio, the number of trays in the absorber unit, the circulation rate of the lean solution, and the operating pressure of the regenerator unit and their influences on the efficiency of the purification process.

### Input temperature of the lean solution

**Fig. 2:**
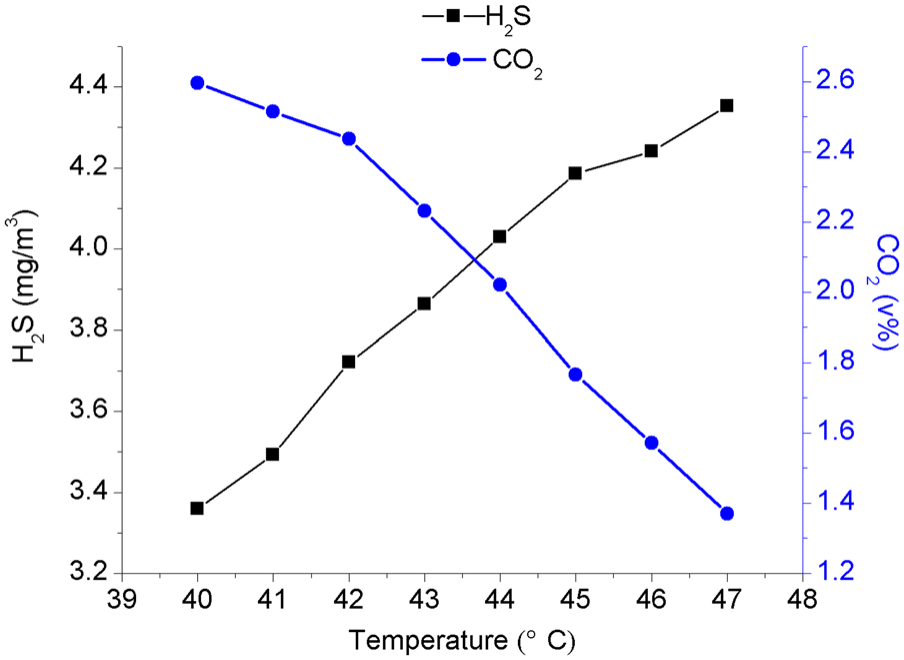
The relationship between the input temperature of the lean solution and the efficiency of the purification process

The purpose of the simulation calculation was to obtain an optimum input temperature for the lean solution while the other operating conditions remained unchanged. The simulated results using the input temperature of the lean solution as a variable are shown in Fig. 2.

The results shown in Fig. 2 revealed that an increase in the input temperature of the lean solution caused a slight increase in the H_2_S content and a continuous decrease in the CO_2_ content in the wet purified gas, as well as a decrease in the flow rate of the wet purified gas leaving the tower. Based on this trend, a decrease in the input temperature of the lean solution reduced the CO_2_ content significantly while the H_2_S content remained almost constant. This result demonstrated that the input temperature of the lean solution primarily affected the absorption rate of CO_2_, while the MDEA-DEA mixed-amines solution did not significantly affect the absorption rate of H_2_S when the operating temperature was lower than 47 °C.

### Reflux ratio

**Fig. 3:**
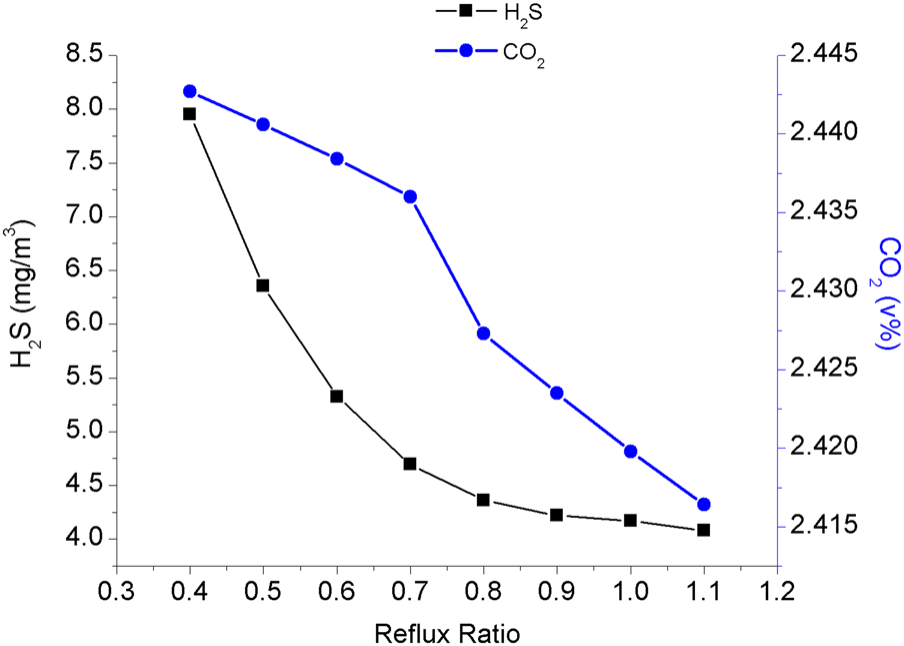
The relationship between the reflux ratio of the condenser and the efficiency of the purification process

The molar ratio between the water dissolved in the acid gas and the desorbed acid gas leaving the regenerator unit is known as the reflux ratio (mol water/mol sour gas). In the simulation calculations, the reflux ratio of the condenser was a variable and the input temperature of the lean solution was set at 42 °C, while other operating conditions remained unchanged. The simulated results are shown in Fig. 3.

Figure 3 shows that the content of the acid gas in the wet purified gas began to level off after the reflux ratio of the condenser reached 0.8 (acid gas load of the lean solution was 0.0029). This revelation illustrated that the influence of the reboiler load on the acid gas load in the rich solution became smaller when the desorption of the acid gas in the rich solution reached a certain level, indicating the effect of the absorption of acid gas after this point became less significant even though the reflux ratio continue to increase.

### Number of trays in the absorber unit

**Fig. 4:**
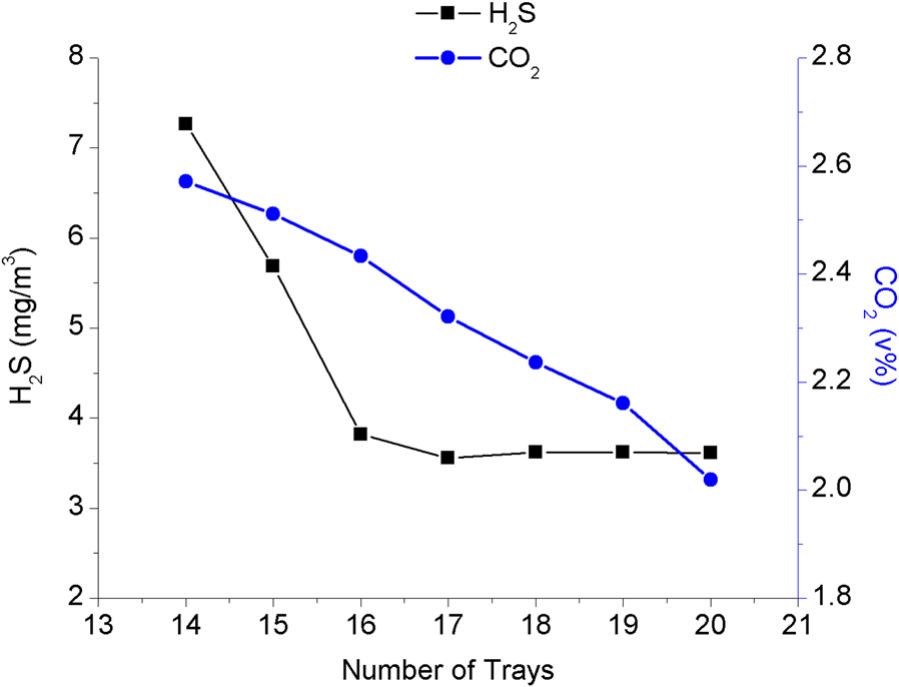
The relationship between the number of trays in the absorber unit and the efficiency of the purification process

Before conducting the simulation calculations, the input temperature of the lean solution and reflux ratio of the condenser were set at 42 °C and 0.88, respectively. The number of trays in the absorber unit was set as the variable to investigate its influences on the quality of the purified gas. The relationship between the number of trays in the absorber unit and the H_2_S and CO_2_ contents in the wet purified gas is shown in Fig. 4.

Figure 4 shows that the content of CO_2_ and H_2_S decreased with the increasing number of trays in the absorber unit. If most of the CO_2_ gas and trace amounts of H_2_S gas were absorbed by the MDEA-DEA mixed-amines solution when the number of trays was increased to 17 trays. The selectivity of the amine solution decreased with the increasing number of trays and caused a substantial amount of CO_2_ to be absorbed. As a result, the flow rate of the wet purified gas dropped accordingly, causing a drop in the revenue of the apparatus and the acid gas quality in the regenerator unit. Therefore, the optimum number of trays in the absorber unit was 17 trays theoretically.

### Circulation rate of the lean solution

**Fig. 5:**
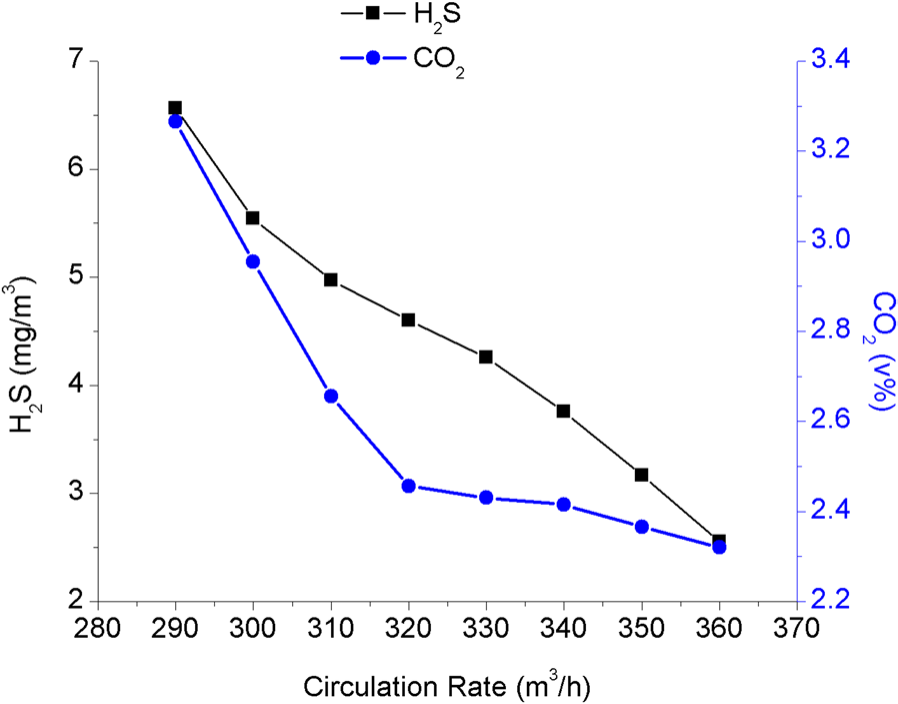
The relationship between the circulation rate of the lean solution and the efficiency of the purification process

Based on previous optimized parameters, the number of trays in the absorber unit, the reflux ratio of the condenser at the top of the regenerator unit, and the input temperature of the lean solution were set at 17 trays, 0.88, and 42 °C, respectively, while the other operating parameters remained unchanged. The step size of the circulation rate was calculated as 10 m^3^/h using the circulation rate of the lean or semi-lean solution as a variable in the simulation calculations. The simulation software was also used to estimate the influence of the circulation rate of the lean solution on the desulfurization and decarbonisation processes. The influence of the circulation rate of the lean solution on the desulfurization and decarbonisation processes is shown in Fig. 5.

The contents of CO_2_ and H_2_S in the wet purified gas decreased with the increasing circulation rate of the lean solution. However, the CO_2_ absorption rate eventually dropped when the circulation rate of the lean solution was increased beyond 320 m^3^/h. This was because the reaction rate between CO_2_ and MDEA decreased. To remove a large amount of CO_2_, the retention time of the lean solution on the tray had to be maximized. Thus, increasing the circulation rate inevitably shortened the retention time of the lean solution on the tray, which slowed down the CO_2_ absorption rate. In addition, the reboiler load increased with the increasing circulation rate. Based on the simulation results, the circulation rate of the lean solution was estimated to be 330 m^3^/h when the design margin of the apparatus was taken into account.

### Operating pressure of the regenerator unit

**Fig. 6:**
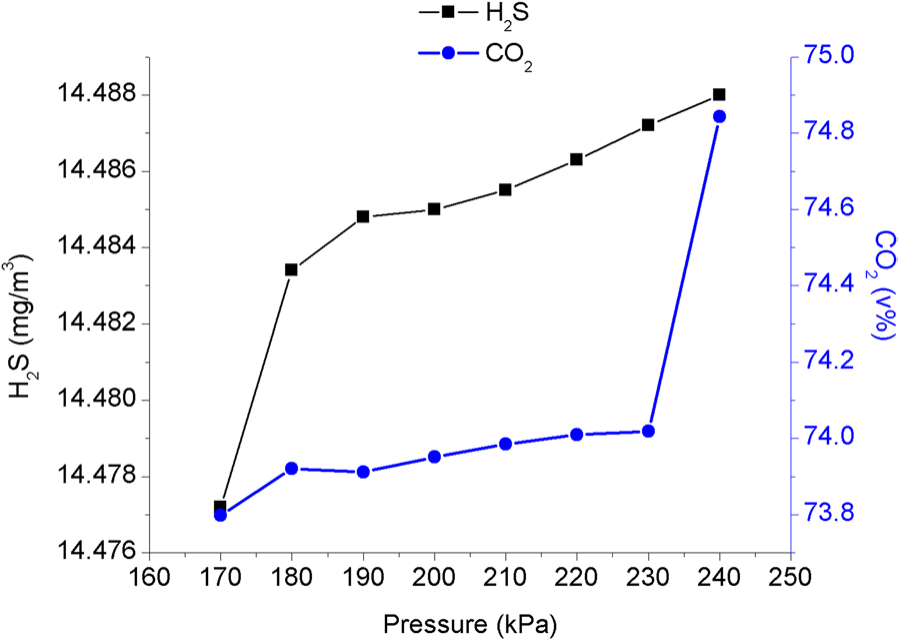
The relationship between the operating pressure of the regenerator unit and H_2_S and CO_2_ contents in the sour gas

Based on previous optimized parameters, the number of trays in the regenerator unit, the number of trays in the absorber unit, the circulation rate of the lean solution, and the reflux ratio were set at 25 trays, 17 trays, 330 m^3^/h, and 0.88, respectively. The simulation results using the operating pressure of the regenerator unit as a variable are shown in Fig. 6.

As shown in Fig. 6, the CO_2_ content increased and the H_2_S content did not change much when the operating pressure of the regenerator unit increased. As a result, the quality of the acid gas degraded. Due to the operating pressure of the regenerator unit increasing almost proportionally with the reboiler load (Wen et al. 2007), the operating pressure should not be too high. Combining the simulation and experimental results, it was more appropriate to set the operating pressure of the regenerator unit at 200 kPa.

## Conclusions

Based on previous analyses of the optimization of inlet temperature of the lean solution, reflux ratio of the condenser at the top of the regenerator unit, number of trays of the absorber unit, circulation rate of the lean solution, and operating pressure of the regenerator unit, a comparison was made on the data before and after the optimization of the apparatus used for the desulfurization and decarbonisation process employing the MDEA-DEA mixed-amines absorption method, as shown in Table 7.

**Table 7:** A comparison of the parameters before and after the optimization

Contents		Before optimization	After optimization
Circulation rate of lean solution, m^3^/h		330	330
flow rate of the wet purified gas, 10^4^Nm^3^/h		185	184.9
Wet purified gas			
H_2_S, mg/m^3^		3.7216	4.2562
CO_2_, %		2.4359	2.4307
Heat load of reboiler, kW		29,180	28,980
Acid gas load of rich solution, mol/mol		0.5062	0.5006
Electric energy consumption, kWh/d		20,467	19,546
0.4Mpa flow rate of vapor, t/d		927	920
Recycled chilled water, t/d		6415	6402
Supplementary deoxygenation water, t/d		47.4	45.2

The flow rates of the wet purified gas remained almost the same before and after optimization. The revenue of the sales gas changed slightly before and after optimization. However, energy consumption of the apparatus, vapor flow rate, flow rate of the recycling cooled water, and the flow rate of the deoxygenated water decreased to different extents. The total costs of energy consumption were reduced. The reboiler load was reduced to a certain extent. Additionally, the acid gas load of the rich solution also dropped slightly, decreasing the probability of causing excessive corrosion of the apparatus and pipe materials. Based on the simulation of the deacidification of the natural gas produced by the Oudeh gas field, the conclusions of the optimization were as follows:

1. When the inlet temperature of the lean solution was less than 47 °C, the CO_2_ absorption rate was mainly governed by the temperature of the lean solution, while the MDEA-DEA mixed-amines solution had less effect on the H_2_S absorption rate.
2. The influence of the reboiler load on the acid gas load in the rich solution became smaller when the desorption of acid gas in the rich solution reached a certain level. For instance, after the reflux ratio of the condenser reached 0.8, absorption of acid gas became less significant, even though the reflux ratio continued to increase.
3. The selectivity of the amine solution decreased with the increasing number of trays and caused a substantial amount of CO_2_ to be absorbed. As a result, the flow rate of the wet purified gas dropped accordingly, causing a drop in the revenue of the apparatus and the sour gas quality in the regenerator unit.
4. The CO_2_ and H_2_S contents in the wet purified gas decreased with the increasing circulation rate of the lean solution. However, the CO_2_ absorption rate eventually decreased when the circulation rate of the lean solution exceeded a certain level (320 m^3^/h).
5. The CO_2_ content increased and the H_2_S content did not significantly change when the operating pressure of the regenerator unit increased. As a result, the quality of the sour gas was degraded. Therefore, the operating pressure should not be too high.
